# Challenges in public health and epidemiology research in humanitarian settings: experiences from the field

**DOI:** 10.1186/s12889-020-09851-7

**Published:** 2020-11-23

**Authors:** Debarati Guha-Sapir, Sarah Elizabeth Scales

**Affiliations:** 1 Centre for Research on the Epidemiology of Disasters (CRED), Institute for Health and Society, Clos Chapelle-aux-Champs, University Louvain, Bte B1.30, 15 1200 Brussels, Belgium; 2 Mailman School of Public Health, Columbia University, 722 W 168th St, New York, NY 10032 USA

**Keywords:** Epidemiology, Humanitarian settings, Disasters, Natural hazards

## Abstract

**Background:**

Humanitarian settings often present unique scientific challenges and conditions that distinguish them from standard research settings. While a number of these challenges are faced in both standard settings and humanitarian settings, factors unique to humanitarian settings such as inaccessibility and time sensitivities further exacerbate the effects of these challenges. This analysis focuses on experiences in post-disaster contexts such as Indonesia and India following the 2004 Indian Ocean Tsunami, the Philippines following Typhoon Haiyan in 2013, and Nepal following the 2015 earthquake.

**Discussion:**

Particular issues that we faced in undertaking research in post-disaster settings include challenges with uncharted ethical and cultural considerations, non-standardised administrative methods for record keeping, data sharing and dissemination. While these issues are not unique to post-disaster humanitarian settings, the time-sensitive nature of our work exacerbated the effects of these concerns. Relying on local partners and making quick decisions to tackle issues is imperative for navigating both foreseen and unforeseen challenges. While pre-emptive action to address these concerns is the most efficient means to expedite research protocols, adaptability and contingency planning are key components of practical research implementation in dynamic situations.

**Conclusions:**

Research is not always a priority in humanitarian settings, so innovative methods are necessary to conduct meaningful and situationally appropriate research in these venues. By understanding available resources, local culture, and political considerations and working efficiently and decisively, we can begin to jump hurdles associated with epidemiologic research in humanitarian settings.

## Background

Since 2000, climate disasters have accounted for roughly 90% of the 7345 disasters recorded in EMDAT [1, 2]. Floods have occurred 5 times as frequently in the last decade compared to 1980s with over 3000 major events since 2000. As more humanitarian crises are precipitated by disasters, health research in humanitarian settings must continue to grapple with these complex environments. While studying human impact and deaths is of utmost importance, these studies also generate questions related to the ethics of such activities [3]. Further, the conditions in post-disaster settings present hurdles to research that must meet acceptable scientific standards while also navigating unusual barriers to implementation. While humanitarian settings characterised by armed civil conflict share similarities with those following disasters, they present unique challenges such as higher levels of rebel or government resistance and elevated risk for both researchers and respondents. Acknowledging these differences, we focus on lessons we have learned in disaster settings that can serve to improve the quality of future research.

In this paper, we focus on three important lessons learned primarily from four significant studies in three major disasters: 2004 Indian Ocean tsunami, Indonesia and India [4–6]; 2013 Typhoon Haiyan, Philippines [7, 8]; and 2015 earthquake, Nepal [9]. These experiences have proved the most challenging and highlighted the need for methodological or organisational solutions for improved quality of results in the future studies.

## Main text

### Importance of standardised methods and quality of data reporting

Methods for data collection are a key component of reliable, publishable results and are dependent on the type of study undertaken. Many epidemiological studies use household surveys to interview victims of the disaster to assess effects such as mortality, malnutrition, mental health, vaccine-preventable diseases, and access to care. Among these, mortality is one of the most useful indicators of impact in humanitarian emergencies, but it has been challenging to measure with convincing accuracy [10–12]. Such poor understanding of death tolls and risk factors compromises effective preparedness and prevention programmes.

A fundamental problem that has not been satisfactorily resolved is representative sampling of affected populations. Mass displacement and inability to define sampling frames complicates implementing household surveys. Two-stage cluster sampling circumvents the need for household lists, but this method presents weaknesses such as establishing denominators, obtaining an acceptable sampling frame, and keeping design effects within acceptable limits. In disaster settings, morbidity and mortality often occur in clusters, so design effects can balloon, weakening the quality of results and associated findings. Further, deaths are typically presented in global totals as opposed to age and gender differentiated mortality. More nuanced information on deaths would allow for the establishment of specific risk factors leading to death and disability while giving more empirical grounds for developing local preparedness policies. Working toward this objective, we chose to use data from health facilities.

The 2004 Indian Ocean tsunami devastated Indonesia and India ultimately causing damage across 18 countries. Nearly a quarter of a million people were killed, including roughly 10,000 Indians who were killed in a matter of minutes. We conducted studies to understand the risk factors for mortality, injury, and epidemic-prone diseases post-tsunami in Jakarta and Banda Aceh, Indonesia and in Tamil Nadu, India while working alongside the Voluntary Health Association of India (Tamil Nadu), a nationwide network of emergency health response, and the International Committee of the Red Cross (ICRC) in Aceh, Indonesia.

In Tamil Nadu, a government compensation scheme for all tsunami-related deaths was rapidly setup. The head of the Indian team obtained an exhaustive list of all deaths in the district, the current addresses of next-of-kin and the population of the sub-district. We were able to draw a systematic study sample, giving us a statistically robust design and avoiding previously discussed design effects. Age and sex data were available for most deaths, and we were able to produce evidence-based conclusions which informed the State Ministry of Health disaster risk reduction plans.

One of the more important findings suggested that coastal families whose main occupation was fishing and/or related activities were at significantly higher risk of death compared to coastal families engaged in other sectors [6]. Surprisingly, we found that young and middle-aged men in these fishing families were at higher risk for mortality compared to others. Relying only on statistical analyses can be misleading, especially in situations where the data or sampling may be weak. Therefore, we organised focus group discussions to contextualise our quantitative findings. The discussions indicated this was likely attributable to these individuals being the only swimmers in the neighbourhood and dying while attempting to rescue others. These findings provided quality assessments for prevention and preparedness policies in Tamil Nadu. The strengths of this study were the availability of exhaustive lists of deaths and access to families. Weaknesses attributable to faulty sampling or data collection in emergent situations can greatly benefit from qualitative techniques to elucidate the findings.

The use of facility-based data, preferably in the form of patient records, is another approach that allows for analyses by demographic categories, improves data quality, and strengthens the estimation of mortality and morbidity due to disasters. Being able to ascertain objective diagnosis of injuries and cause of death from clinical sources is another advantage of using facility-based data. Self-reported morbidity is often less reliable in terms of diagnostics and recall biases that can reduce the quality of the findings. Civil registration services may not be functional after natural disasters and often take considerable time to be reinstated. When reinstated, the registration is rarely retroactive and statistical black holes remain. Large-scale displacement, destruction of roads and access channels, and whole-family deaths are factors that complicate population sampling methods and make the use of facility data a defensible choice.

We have undertaken four studies using facility-based data complemented by qualitative techniques. Facility-based data usage has its limitations, but given the context of our studies, the strengths of using these data outweighed the potential selection bias. These designs were developed after initial preparatory work indicated that population-based sampling presented too many obstacles. We characterised changes over time in the child/adult ratio of consultations and whether post disaster morbidity differed significantly from pre-disaster patterns. In the Indonesian study, we collected data from the ICRC field hospital, which was the first facility functional from day 3 of the event [4]. We analysed diagnostic data from hospital records including key demographic information on each patient. Using hospital data conferred a greater level of scientific validity to the study and established more reliable morbidity profiles. This allowed an analysis that revealed trends in the evolution of pathologies and the changes in the age-sex patterns among victims. Incompleteness and illegibility of patient logs in the first crucial days of rapidly deployed field hospitals present additional barriers. In Aceh, we frequently faced data challenges with records from the first 48 h of hospital operation where many logs were unreadable or inconsistent (e.g. future dates or male pregnant). For our study, doctors and nurses on duty in the early days of response were still onsite when we arrived, and we were able to painstakingly recreate the individual records through interviews with the attending medical staff. Admittedly, this process is not a sustainable method to address the persistent problem of incomplete facility-based records. We found that the sooner the team is on site and operational, the better the final likelihood of high-quality data.

Based on our success with the ICRC hospital in Aceh, we designed two further studies: one following the earthquake in Nepal and another after Typhoon Haiyan in the Philippines. Focusing on disaster-related morbidity patterns across demographic and diagnostic groups, we examined the pattern of injuries from hospital records in the post disaster period and subsequently compared them to the same period the year before the disaster [8, 9]. We worked with a medical researcher from the Philippines who was trained at CRED and with our partner hospital medical director in Nepal. In both studies, we established detailed cooperative agreements with the first line hospitals that received the bulk of disaster victims and discussed the research questions that were important for them. Using the facility-based approach again circumvented the major hurdles of obtaining requisite representativeness and limiting recall bias inherent to self-reported injuries. Using hospital data was also logistically beneficial given limited access for research teams to affected areas. Secondly, many of the affected areas were out of reach when field work was undertaken at each site. Hence, the use of hospital records as the main data source, supported by equitable, pre-established agreements with local partners, hospitals, and state authorities, was a key factor for success.

There is little question from our experience that a central consideration to ensure satisfactory completion of studies in post-disaster contexts is to discuss, explain, and consult the local and hospital authorities from the very start. Data quality is central to promising publication outcomes and is worthy of in-depth, realistic assessment before finalising study designs. Detailed preparation with national partners and dry runs of data extraction are critical for sound study implementation. This approach not only improves the study focus but also allows the design to be reworked, taking into account the local constraints which are often unforeseen in a survey or data collection handbook. In the studies discussed here, considerable time and effort was invested onsite in the initial phases of the field work to ensure cooperation and utility of the proposed study aims for future health preparedness and prevention in the affected communities.

### Political / cultural clashes

In addition to methodological hurdles, the politics of death tolls can be a major obstacle to correct reporting. While this is of overwhelming importance in armed civil conflicts where deaths become the main driver of the conflict and international intervention, it has recently become an issue even for natural disasters. For example, following Hurricane Maria hitting Puerto Rico, where the US government, in an effort to minimise the impact and downplay the inadequate response reported an implausibly low death toll [12, 13]. On the other hand, overestimations of the death tolls, often in civil conflicts, can be used to prompt humanitarian assistance. Civil conflict settings provide examples of such controversies. For example, mortality estimation studies of the Darfur massacres were coloured by the genocide case brought against President Omar Basher by the International Criminal Court [14–17]. Natural disaster mortality estimations can learn lessons from conflicts where high mortality is often reported by advocacy groups outraged by killings of civilians contradict lower estimates with using statistical methods. A similar scenario played out in the Iraq War [18, 19] where the deaths tolls varied widely, and the debate pitted scientific quality versus political interests [12]. These difficulties serve to highlight the need for stringent scientific protocols for mortality reporting in all humanitarian crises.

Local politics plays an important part not only in the implementation of the study but also in the dissemination of its findings. Politics is at the heart of discussions of humanitarian aid and infiltrates discussions of medicine and health more broadly. But the political context should not overshadow the objective of bettering the health and wellbeing of affected populations. Understanding the political dynamics of the disaster setting is important to undertaking meaningful research and getting the results out in time (and sometimes, altogether).

In our experience, political overtones were particularly present in the study of Typhoon Haiyan, where civil society sources reported higher numbers of deaths than state estimates. The implication of this discrepancy pointed a finger at the state’s inadequate response which, the civil society organisations claimed, led to higher numbers of deaths. This is a good example of the political tensions that may arise from otherwise innocuous death tolls from disasters. Political tensions can only be sustainably addressed through a balanced and sound representation of a study’s findings and laying out its limitations completely and accurately. Scientific credibility is the strongest defence researchers have and is most likely to maintain working relationships for potential future studies. Overt engagement in political positioning for scientific publications is likely to have negative consequences both in the field and later in the wider reception of the scientific community. We have found that while there are often political factors that shadow the context, it may be best to leave political analyses to specialists who are better equipped to do so.

### Uncharted ethical waters and reciprocity

Post-disaster research often has to be conducted on a tight timescale to be useful for policy change. Ethical considerations in these situations are paramount for several reasons and must be respected. But while on one hand speed in getting the research results out is unquestionably important, delays in IRB approvals [20], especially for alterations to the initially approved protocol, can prove to be very time consuming. Therefore, researchers working in disaster contexts have a “duty to plan,” pre-emptively taking into consideration the approvals that will be required [21]. Effective planning includes anticipating impediments related to ethical issues for research implementation, including pre-emptive mitigation measures for such situations.

In the Philippines, using hospital records required many unexpected layers of internal clearances which, while legitimate, substantially delayed the start of data extraction and increased costs. Entities that had the authority to clear the research protocols had other concerns of higher priority at the time than pushing through our research approvals. Likewise, staff and their families were themselves victims, and staff attrition became a major obstacle. Any research effort that the staff provided implied time away from work or their personal needs [4]. This is an important concern for both researchers and the subjects. We faced some serious moral dilemmas with regard to engaging time of the victims or local staff for research participation at times of stress and survival. Competing needs and priorities of local personnel are both moral and practical dilemmas in the field and need to be foreseen to minimise negative effects.

Differences in cultural practices and norms exist between international, national, and within-country teams, especially when external actors enter the affected zone. In these studies, we have found that views, however well-intentioned, can be fundamentally different between international, globally experienced teams and local collaborators. After 3 weeks of working with a local translator following the tsunami in Aceh, we learned she had lost both her small sons in the disaster. She explained that in her culture sharing intimate grief and loss with strangers was difficult, preferring to keep such grief within her family or members from her own community. Although this did not affect our research, it did reveal that cultural differences, even if personal in nature, should be kept in mind.

In this context, local partners are invaluable resources for learning about and understanding the affected populations’ cultural and societal norms. In Nepal, we had strong, professional partners with whom candid discussions of ways to overcome snags were possible on both sides. Successful research in post-disaster settings requires careful attention to the social implications and power structures of the study community. For example, key informant interviews or focus group discussions could be sensitive to local power dynamics, such as clinician-patient or other social hierarchies [20]. In Tamil Nadu, where the caste system still plays an important role, intra-caste groups had to be formulated to minimise undue influence of any one member. Comprehensive review of cultural, moral, and ethical implications of research participation by local partners before finalising the research protocol is a worthwhile investment.

Humanitarian emergency response today involves over 30 billion USD annually, not including national contributions from the affected countries. At such a high price tag, evidence-based scientific research underlies ethical allocation of resources and implementation of programs. Conducting research to improve the quality of response is widely acknowledged and much is being done to expand evidence and generate and share data to support sound and effective policy [22, 23].

First, given the complicated nature of post-disaster research, open data should be utilised as an avenue to bolster the work of the wider research community. Our investigation and the work of others could have benefitted from stronger comparative analyses made possible through access to other datasets. That said, data is increasingly shared either at the request of journals or voluntarily by researchers themselves. The UN Office for the Coordination of Humanitarian Affairs (UNOCHA) has set up the UN Humanitarian Data Exchange (HDX) with 17,751 available datasets from 253 locations [24]. Similar scientific data platforms would undoubtedly ensure faster progress, stronger results, and more equitable access for many researchers in both developed and developing countries.

Secondly, delays in the release of research results are barriers to timely action, a central concern and ethical imperative in humanitarian crises. The results from the tsunami studies [4–6] were shared within a few months from the initiation of work, and both influenced disaster preparedness and prevention policies. But after Typhoon Haiyan, tensions resulting from the discrepancy between civil organisations and other authorities could have affected the dissemination of our research. While these tensions did not impact release, the entire research undertaking was significantly delayed due to the tedious clearance processes. Timely sharing of results and reciprocity are key for providing the scientific foundation for humanitarian response.

## Conclusions

There are a number of important lessons from the obstacles we have faced in undertaking field research after disasters. Contingency plans for dealing with logistical and administrative challenges help to anticipate potential barriers and foster timely problem solving for those that are unforeseen. Choosing data collection methods and sources to sidestep concerns associated with displacement and destruction ensures access to study subjects. It also mitigates concerns of bias introduced into standard sampling frames used in population surveys.

Research teams must work closely with local teams, consult with them from the start, and share research benefits fairly. This is not only in the interests of equity, but it protects the research outputs from embarrassing oversights and enriches the results. Further, researchers in humanitarian settings must weave through complex political and cultural labyrinths, requiring a clear idea of the agendas being promoted by different stakeholders and the potential impact these narratives have on the dissemination of research. Understanding cultural and circumstantial factors surrounding research, disseminating results in a timely manner, and sharing data are critical for the ethical conduct of research amongst vulnerable, disaster-affected populations.

For our work, the epidemiologic and demographic characterisation of morbidity and mortality immediately following disasters substantively adds to the field of disaster epidemiology. All researchers must continue to press forward in undertaking this critical work. Surmounting hurdles associated with work in humanitarian settings allows timely and credible application of research to policies and practices.

## Data Availability

No datasets were used for the purposes of this study.

## Abbreviations

CRED: Centre for Research on the Epidemiology of Disasters
EM-DAT: Emergency Events Database
IRB: Internal Review Board
ICRC: International Committee of the Red Cross
TUTH: Tribhuvan University Hospital
HOPE: Hospital Preparedness for Emergencies
SFDRR: Sendai Framework for Disaster Risk Reduction
UNOCHA: United Nations Office for the Coordination of Humanitarian Affairs
HDX: Humanitarian Data Exchange
